# Persistent Fever and Cough in a Patient With Good’s Syndrome: A Case Report

**DOI:** 10.7759/cureus.24996

**Published:** 2022-05-14

**Authors:** Anwar S Turaes, Weaam K Alharbi, Raghad K Alqurashi, Abdulfattah Touman, Adeeb Bulkhi

**Affiliations:** 1 Medical School, Umm Al-Qura University, Makkah, SAU; 2 Pulmonology Department, King Abdullah Medical City, Makkah, SAU; 3 Department of Internal Medicine, Umm Al-Qura University, Makkah, SAU

**Keywords:** good’s syndrome, hypersensitivity pneumonitis, thymoma, hypogammaglobulinemia, adult-onset immunodeficiency

## Abstract

Good’s syndrome is a rare, acquired immunodeficiency condition characterized by thymoma and hypogammaglobulinemia, which increases the risk of recurrent infections. Immunoglobulin replacement therapy (IgRT) is the key treatment for recurrent infections.

We describe the case of a 57-year-old male with a history of an anterior mediastinal mass and a persistent cough lasting for a few years. Based on the clinical history and immunological analysis, he was diagnosed with Good’s syndrome. He was being managed conservatively with immunoglobulin until he underwent a thymectomy. Subsequently, he developed his first pneumonia. His conditions gradually worsened despite the initiation of IgRT. He was diagnosed to have hypersensitivity pneumonitis based on strong exposure history, consistent radiological images, and good clinical response to antigen avoidance and steroid therapy.

To our knowledge, this is the first case of Good’s syndrome with hypersensitivity pneumonitis that was unmasked after immune augmentation by the initiation of IgRT. Moreover, surgical intervention should not be considered unless unavoidable. Additionally, close clinical monitoring and laboratory testing are indicated, and IgRT should be considered when the patient begins to exhibit symptoms to prevent severe infections.

## Introduction

Good’s syndrome is a rare, acquired immunodeficiency condition that occurs in people aged 50-70. There are only 0.15 cases of Good’s syndrome reported per 100,000 population annually, with fewer than 200 cases reported globally [1]. It is characterized by thymoma and hypogammaglobulinemia, which increases the risk of recurrent infections with encapsulated bacteria, opportunistic viruses, and fungi. Furthermore, patients with Good’s syndrome often have autoimmune conditions, such as myasthenia gravis (30-50%) and idiopathic thrombocytopenia [2].

The pathogenesis of Good’s syndrome remains unknown, with little evidence indicating that bone marrow defects cause impaired maturation of myeloid and erythroid precursors and pre-B cell arrest [3]. Patients with Good’s syndrome usually present with symptoms due to recurrent infection or secondary to the thymoma itself, such as cough, chest pain, dysphagia, dyspnea, and hoarseness. The superior vena cava syndrome, Horner syndrome, and masses in the neck have been reported as initial manifestations of this disorder [3].

Patients with Good’s syndrome usually have few to no B cells in the peripheral blood, abnormal CD4+:CD8+ T cell ratio, and decreased CD4 T cells, and almost all patients have reduced levels of serum immunoglobulin (Ig)G, IgA, and IgM [4]. Occasionally, Good’s syndrome is associated with hematological disorders, with anemia present in over 50% of patients. Pure cell aplasia, aplastic anemia, hemolytic anemia, and pernicious anemia have all been described in patients with Good’s syndrome [5]. The most common infection reported is cytomegalovirus (CMV) [6].

The primary management of Good’s syndrome involves thymoma removal and maintenance of IgG levels by Ig replacement therapy (IgRT) to decrease infections and reduce hospitalizations [4]. Most patients with Good’s syndrome are managed as outpatients, with a few requiring hospitalization in case of severe infection [6]. The thymoma associated with Good’s syndrome usually has a benign spinodal histological shape. There is no evidence to support the restoration of immune function after thymoma removal [7]. Thus, surgical removal of thymoma should be considered only if there is a sign of invasion or a threat of obstruction.

Long-term prognosis and clinical outcomes depend on the severity of infection, associated hematologic disease, and the presence of any other autoimmune or immunodeficiency disease [4].

Here, we discuss a case of a patient with Good’s syndrome who presented with cough as the primary symptom and was found to have hypogammaglobulinemia with B cell lymphopenia that was complicated with persistent fever post-thymoma removal.

## Case presentation

We describe the case of a 57-year-old male who suffered from a persistent cough for a few years. The patient was treated for a cough variant asthma at another hospital, with minimal improvement with inhaled corticosteroid/long beta-agonist inhaler. In addition, he had sneezing, an itchy nose, and itchy eyes for approximately one year. He had no apparent history of recurrent sinopulmonary infection or meningitis. He had a history of benign prostatic hypertrophy and an unresected slowly enlarging mediastinal mass for the past 20 years.

A blood serum test showed low hemoglobin levels (11.0 g/dL), low red blood cell count (3.61 × 10^12^/L), and low hematocrit levels (32.5%). The white blood cell count was 5.81 × 10^9^/L, and the platelet count was 275 × 10^9^/L (Table 1).

**Table 1:** Complete blood cell count with differentials in June 2020. CBC: complete blood count; MCV: mean corpuscular volume; MCH: mean corpuscular hemoglobin; HGB: hemoglobin; RDW: red cell distribution weight

CBC with differential	June 2020	Reference range
WBC	5.81 × 10^9^/L	4–11
RBC	3.61 × 10^12^/L	47–60
HGB	11.0 g/dL	13.5–17.5
Hematocrit	32.5%	39–52
MCV	90.0 fL	80–98
MCH	30.5 pg	-
Mean corpuscular HGB	33.8 g/dL	31.9–35.2
RDW	15.7%	11.5–15.3
Platelet	275 × 10^9^/L	140–450
Mean platelet volume	9.4 fL	8.6–12.3
Neutrophil	2.73 × 10^9^/L	2–7.5
Lymphocyte	1.83 × 10^9^/L	1.3–3.5
Monocyte	0.70 × 10^9^/L	0.2–0.8
Eosinophil	0.49 × 10^9^/L	0–0.5
Basophil	0.06 × 10^9^/L	0–0.1

A careful evaluation of serum Ig levels revealed undetectable IgA and IgM and low IgG. Furthermore, the flow cytometry analysis for lymphocytes showed deficient CD19+ B cells, low normal CD4 T cells, low CD4/CD8 ratio, and normal CD8+ T cells and natural killer (NK) cells (CD16/CD56) (Tables 2, 3). Antibody titers to diphtheria and tetanus were normal; however, pneumococcal titers remained non-protective even after unconjugated pneumococcal vaccination.

**Table 2:** Absolute values of immunoglobulin levels in June and December 2020.

Blood test	June 2020	December 2020	Reference range
Immunoglobulin G (IgG)	604 mg/dL	620 mg/dL	700–1,600
Immunoglobulin M (IgM)	18 mg/dL	0.18 mg/dL	40–230
Immunoglobulin A (IgA)	10 mg/dL	0.07 mg/dL	70–400

**Table 3:** Flow cytometry analysis results for lymphocytes in June and December 2020.

	June 2020	December 2020	Reference range
Lymphocyte absolute count	1.382 × 10^3^/µL	2.261 × 10^3^/µL	0.9–3.1
T-lymphocyte (CD3^+^)	1,247 cells/µL	2,000 cells/µL	570–2,400
T-lymphocyte (CD3^+^)	90% lymph	88% lymph	62–87
T-helper cell (CD3^+^/CD4^+^)	465 cells/µL	665 cells/µL	430–1,800
T-helper cell (CD3^+^/CD4^+^)	34% lymph	29% lymph	32–64
T-suppressor cell (CD3^+^/CD8^+^)	699 cells/µL	1,197 cells/µL	210–1,200
T-suppressor cell (CD3^+^/CD8^+^)	51% lymph	53% lymph	15–46
B-lymphocyte (CD19^+^)	22 cells/µL	19 cells/µL	91–610
B-lymphocyte (CD19^+^)	2% lymph	1% lymph	6–23
NK cells (CD16^+^/CD56^+^)	86 cells/µL	194 cells/µL	78–470
NK cells (CD16^+^/CD56^+^)	6% lymph	9% lymph	4–26
CD4^+^/CD8^+^ ratio	0.67	0.56	More than 0.9

**Figure 1:**
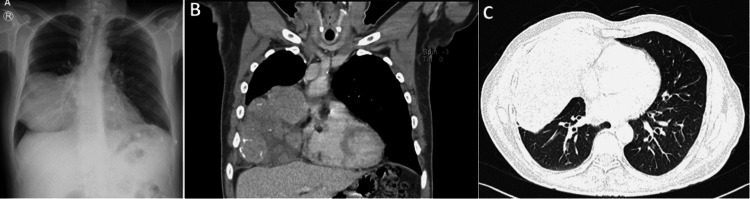
(A) Large well-defined mass silhouetting the right cardiac border extending to the lateral chest wall without invading it. (B) Coronal reconstruction, the soft tissue window showing the internal heterogeneity of the mass with areas of calcifications, measuring 9 × 15.5 × 11.1 cm in AP × TR × CC dimensions, respectively. (C) Axial cut of the lung window showing normal lung parenchyma. AP: anteroposterior; TR: transverse; CC: craniocaudal

Chest X-ray and computed tomography (CT) scan showed a large right chest mass extending from the mediastinal reaching the pleura. The mass had inhomogeneous consistency and showed an area of calcifications. There was no sign of mediastinal nor thoracic wall invasion. Importantly, the lung parenchyma showed no evidence of acute or sequelae of old infections (Figure 1).

Based on his clinical history and immunological analysis, Good’s syndrome was diagnosed. Initially, the patient was managed conservatively with symptom-controlling treatments. Moreover, close monitoring of Ig levels and immediate treatment of infections were considered.

The patient’s cough worsened, and he had progressive trepopnea that progressed slowly over the past year and affected his sleep significantly; therefore, he accepted the surgical excision of the mass. Histopathology showed Type AB thymoma, with microscopic transcapsular invasion, and modified Masaoka Stage IIa.

The immediate postoperative course was complicated by pneumonia that required a course of antibiotics therapy, which covered the pan-sensitive* Haemophilus influenza* which was cultured from his sputum three months earlier during a surveillance sputum culture. *Haemophilus influenza* was not treated at that time as he was asymptomatic, and his chest examination was normal. He received his first IgRT infusion to prevent recurrent infections and optimize his immune status eight months after the surgery.

Three days post-discharge, he reported worsening cough, low-grade fever, and sputum production. Chest examination revealed diminished breathing sound over the right lower lung zone and scattered expiratory wheeze. He was treated for community-acquired pneumonia and administered azithromycin and cefuroxime axetil. His fever and sputum production improved; however, he continued to have a nagging cough, for which he received an ipratropium bromide inhaler and later inhaled corticosteroid with a long-acting beta-agonist without response.

**Figure 2:**
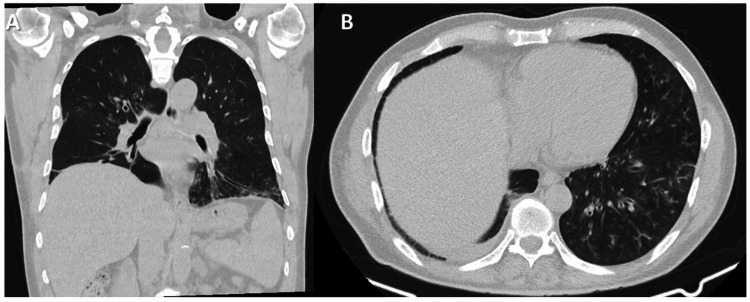
(A and B) Coronal and axial chest CT show centrilobular ground-glass nodules, tree in buds, and thickened airways mainly at the left lower lung lobes.

A follow-up high-resolution CT chest (HRCT) a few weeks later was done due to persistent cough and fever. It showed evidence of airway thickening, centrilobular nodules, and a tree in bud appearance in the contralateral left lung (Figure 2).

At this stage, a proton pump inhibitor was added to his therapy without significant symptomatic response. He continued to receive IgRT; however, another episode of chest infection occurred which was associated with high-grade fever and significant bronchospasm and for the first-time oxygen desaturation.

Bronchoscopy was performed, and an extensive panel of investigations sent from the bronchoalveolar lavage (BAL) included respiratory viral multiplex polymerase chain reaction (PCR), Ziehl-Neelsen staining, *Mycobacterium* PCR and culture, fungal culture, and *Aspergillus galactomannan* antigen all were negative.

Once again, culture growth of pan-sensitive *Haemophilus influenza* was reported from the BAL. He received an extended course of targeted antibiotics without response as his fever continued.

The BAL cell count and differential showed total white blood cells of 188/mm^3^, 90% monocytes, 6% lymphocytes, and 2% neutrophils. A few days later, his condition deteriorated, and he had a severe cough, high fever, and significant oxygen desaturation, and he was admitted for intravenous antibiotics and further investigation of his condition.

**Figure 3:**
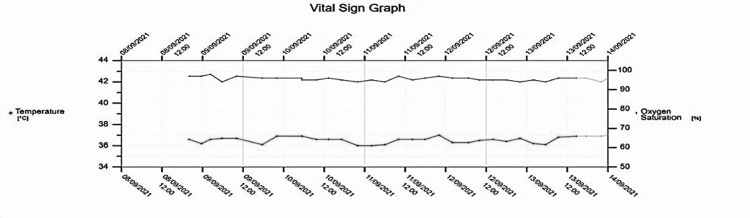
Temperature and oxygen saturation chart showing absence of fever and desaturation during admission to the hospital.

The autoimmune screening which included antinuclear antibodies, rheumatoid factors, and antineutrophilic cytoplasmic antibodies was negative. Pan-CT with intravenous contrast showed no evidence of deep-seated infection. During his hospital course, it was noted that he had no spikes of high temperature nor an episode of oxygen desaturation and his bronchospasm rapidly responded to bronchodilator and steroids (Figure 3).

**Figure 4:**
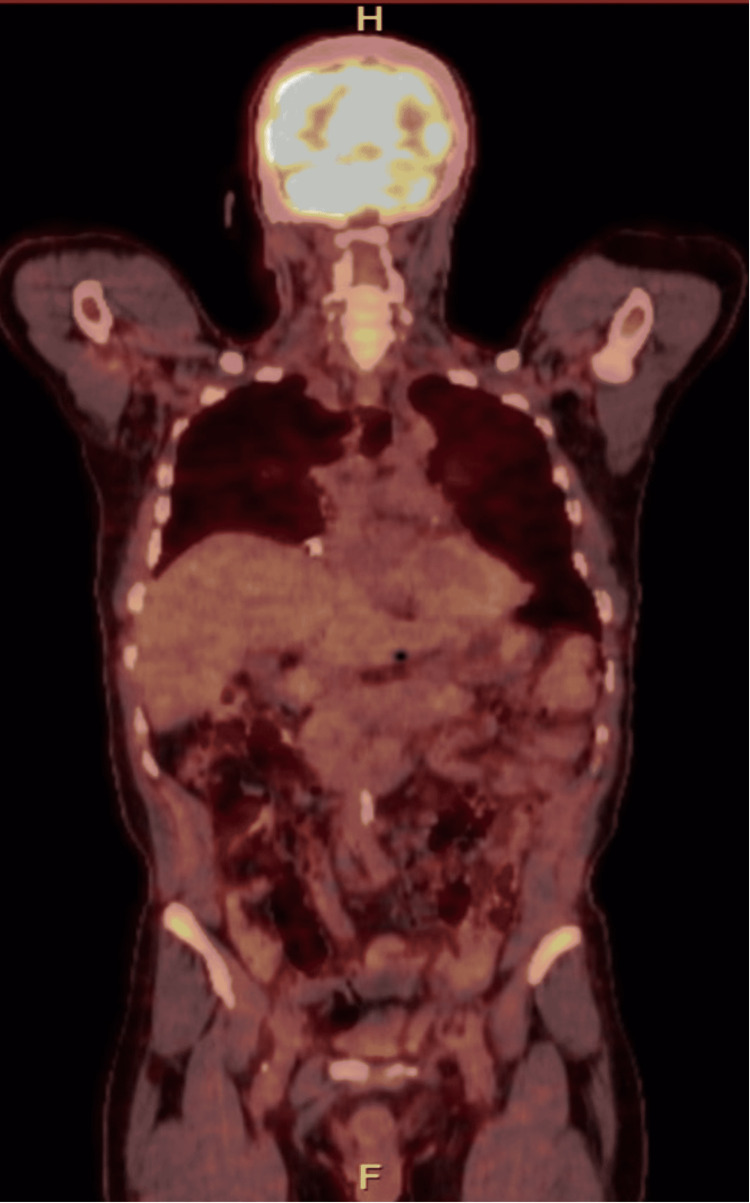
Coronal section of PET-CT scan showing small right lower lobe 0.6 cm nodule with SUVmax 1.3. The described diffusely increased FDG activity of the bone marrow is seen in the iliac bone. PET-CT: Positron emission tomography-computed tomography; SUVmax: maximum standardized uptake value; FDG: fluorodeoxyglucose

Detailed targeted history for potential exposure to an antigen that can trigger hypersensitivity type of reaction revealed that he had a hobby of feeding street pigeons. Based on the compatible HRCT with hypersensitivity pneumonitis (HP). A repeat bronchoscopy with biopsy was offered to solidify the diagnosis; however, he refused it. Cryptococcal antigen test was sent, and the result came negative. Positron emission tomography-computed tomography scan showed small bilateral nodules, the largest in the right lower lobe measuring 0.6 cm with a maximum standardized uptake value of 1.3, and no fluorodeoxyglucose (FDG)-avid uptake in the operative bed. Diffusely increased FDG activity of the bone marrow with no underlying CT changes. No aggressive FDA-avid bone lesions were noted (Figure 4).

The patient’s symptoms improved after antigen avoidance; however, the cough persisted, and, eventually, systemic steroids managed to settle the patient’s persistent cough. He continued IgRT to augment his immunity and reduce the risk of future severe infections.

## Discussion

The patient was diagnosed with Good’s syndrome based on imaging and his consistent immunological profile. He was monitored closely and managed conservatively for years. He preferred not to undergo surgical resection for his slowly growing mediastinal mass until he could not tolerate the mass effect. Unfortunately, after the surgical removal of this thymoma, he suffered from a typical lower respiratory tract infection.

Although he had very low levels of IgA and IgM, his IgG level was relatively higher at 620 mg/dL. Nevertheless, there was no response to pneumococcal vaccination, and his B cell levels were very low. Thus, the patient received IgRT to reduce the incidence of further severe infections. Since the initiation of IgRT, there was a progressive worsening of the pulmonary condition and he started to develop bronchospasm, cough, wheeze, and fever.

Based on the history of a strong association between pigeon antigen exposure and symptom worsening; in addition to consistent radiology and complete resolution of symptoms upon avoidance, hypersensitivity pneumonitis was diagnosed and treated.

The exact contribution of the immune augmentation therapy to a pathophysiological mechanism that caused the HP to exacerbate is yet to be explained. Managing patients with such rare cases is challenging, nevertheless, patients with Good’s syndrome should be closely monitored with at least three to six months of following up Ig and lymphocyte levels if detected early and not presenting with recurrent infections.

In case of low IgG, patients should receive IgRT in continued regular sessions every three months to maintain IgG levels of above 500 mg/dL or every month, if the patient presents with recurrent infections regardless of IgG levels, to reduce the incidence of further severe infections [7]. Therefore, the mainstay of treatment for Good’s syndrome is IgRT because several case reports have shown that it reduces the risk of infections. The most common cause of hospitalization in Good’s syndrome is infections, including pathogenic bacteria and opportunistic pathogens such as CMV and *Pneumocystis jirovecii* pneumonia (PJP) [6]. For this reason, it is recommended for patients with Good’s syndrome to take antibiotic prophylaxis and vaccines, including PJP and herpes simplex virus [8].

To our knowledge, this is the first case of Good’s syndrome with HP that was unmasked after immune augmentation by the initiation of IgRT. Moreover, surgical intervention should not be considered unless unavoidable. Additionally, close clinical monitoring and laboratory testing are indicated, and IgRT should be considered when patients begin to exhibit symptoms to prevent severe infections.

## Conclusions

Good’s syndrome is characterized by hypogammaglobulinemia and reduced or absent B cells. Patients usually present with recurrent infections, such as encapsulated bacteria, opportunistic viruses, and fungi, or symptoms secondary to the thymoma. The main prognostic factor impacting the survival of patients is IgRT to reduce the risk of recurrent infections. Additionally, close clinical monitoring and laboratory testing are indicated. Furthermore, surgical intervention should not be considered unless unavoidable. In patients with Good’s syndrome, the long-term prognosis depends on the severity of the infections. Further research and studies are needed to clarify this syndrome.
